# ANTIPLASMODIAL ACTIVITIES OF THE STEM BARK EXTRACT OF *ARTOCARPUS ALTILIS* FORSBERG

**DOI:** 10.21010/Ajid.v16i2S.5

**Published:** 2022-08-17

**Authors:** Aladesanmi, Adetunji Joseph, Odiba, Ogu Emmanuel, Odediran, Samuel Akintunde, Oriola, Ayodeji Olubunmi

**Affiliations:** † Department of Pharmacognosy, Faculty of Pharmacy, Obafemi Awolowo University, Ile-Ife, Osun State, Nigeria; ® Drug Research and Production Unit, Faculty of Pharmacy, Obafemi Awolowo University, Ile-Ife, Osun State, Nigeria; ⸸ Department of Pharmacognosy and Herbal Medicine, College of Pharmacy, Afe Babalola University, Ado Ekiti, Ekiti State Nigeria

**Keywords:** Antiplasmodial, antimalarial, Chemosuppressive, *Artocarpus altilis* stem bark, Gas Chromatography-Mass Spectroscopy

## Abstract

**Background:**

The potential of *Artocarpus altilis* stem bark as a safe antimalarial agent, and the identification of its antimalarial constituents was explored.

**Materials and Methods:**

The air-dried stem bark was extracted with 70% ethanol, filtered and concentrated *in vacuo* to obtain the extract (**EE**). The extract was successively partitioned to give *n*-hexane (**AAH**), dichloromethane (**AAD**), ethyl acetate (**AAE**) *n*-butanol (**AAB**) and aqueous (**AAQ**) fractions respectively after determining the acute toxicity using Lorke’s method. These were each evaluated for chemosuppressive antimalarial activities (0-200mg/kg) against chloroquine-sensitive *Plasmodium berghei-berghei*-infected albino mice. Normal saline and chloroquine, 10 mg/kg were negative and positive control respectively.The survival times and percentage survivors of the mice in both experiments were determined after observation for twenty-eight days post-drug administration. The five (5) column chromatographic (**CC**) fractions, **AAH1**, **AAH2**, **AAH3**, **AAH4** and **AAH5** obtained from the most active **AAH,** were also evaluated for antimalarial activities (0-50mg/kg). Further column purification and repeated **PTLC** of **AAH5** yielded three bands, which were finally subjected to GC-MS analysis.

**Results:**

**EE** gave ED_50_ and LD_50_ values of 227.17and >5000 mg/kg while its partitioned fractions gave ED_50_ values as follows: **AAH,** 79.14; **AAD**, 215.59; A**AE,** 160.46, **AAB,** .42; and **AAQ**, 90.85 mg/kg respectively. The primary **CC** fractions also gave **ED**_50_ values as follows: **AAH1** 21.95; **AAH2,** 26.96; **AAH3,** 21.30; **AAH4,** 20.92 and **AAH5,** 20.75 mg/kg respectively to identify **AAH5** as the putative fraction. GC-MS analysis revealed eleven major compounds (**1–11**) in the three **PTLC** bands as the antiplasmodial constituents of the plant.

**Conclusion:**

The stem bark of *A. altilis* is a potential agent in malaria control which is safe for oral use.

## Introduction

The alarming incidence of malaria all over the world, particularly in developing countries, calls for concerted efforts towards proffering a lasting solution especially with the development of resistance to the existing therapeutic drugs. The crude extracts of natural products that are administered locally at very low cost for the basic healthcare needs among populations in the developing countries are promising therapeutic candidates in the search for remedies to manage this disease. Hence, the scientific evaluation of natural products used as medicines in the management of malaria is justified (WHO, 1993; Canter *et al.*, 2005).

Malaria which is caused by approximately 200 known species of organisms, belonging to the genus *Plasmodium* (Phylum Apicomplexa) infects particular lineages of primates, rodents, birds, and reptiles. It has been shown to cause malignant sub tertian, benign tertian, benign quartan and a milder form of benign tertian malariae respectively (Mendis *et al.*, 2001; White, 2008), while *P. falciparum* is the most pathogenic, causing high incidence of severe brain involvement and other complications (Bowman and Rand, 1980; Antoniana *et al.*, 2001). The spread of multi-drug resistant malaria parasite strains has highlighted the urgent need for the development of new, inexpensive, affordable, and safe anti-malarial drugs for developing countries where the disease is prevalent (Wernsdorfer and Trigg, 1988; Miller, 1992; Vial, 1996; Bickii *et al.*, 2000).

*Artocarpus altilis* Forsberg of the family (Moraceae) which is commonly called ‘bread fruit’ is a profitable multipurpose medicinal plant possessing useful biological activities. Such activities include, wound healing, expectorant, antidiarrhoeal, antidiabetic, anti-malarial, anti-fever, antisyphilic, antianaemic, antiasthmatic, anthelminthic against tapeworm infections and antidermatitic. It is therefore likely to be an important source of secondary metabolites including antimalarials (Jagtap and Bapat, 2010).

The antitubercular and antimalarial activity of its root has led to the isolation of some prenylated flavones. These include cycloartocarpin, artocarpin and chaplashin obtained from the dichloromethane extract of the root and stems and compounds like morusin, cudraflavone B, cycloartobiloxanthone, artonin E and artbiloxanthone which were found in the root bark. They all exhibited moderate antiplasmodial activity with IC_50_ values ranging from 1.9 to 4.3 μg/ml. (Boonphong *et al.*, 2007, Jagtap and Bapat 2010). The anti-diabetic and antibacterial activity of the root bark (Tsuchiya *et al.*, 1996; Khan *et al.*, 2003; Trindade ***et al.*, 2006**; Adewole and Ojewole, 2007, Suhartati *et al.*, 2008) have also been reported.

The *Artocarpus* species are rich in phenolic compounds including flavonoids, stilbenoids and arylbenzofurons (Hakim, 2010). The dichloromethane extract of the leaf of *A. altilis* have also yielded β-sitosterol, unsaturated triglycerides, squalene, polyprenol, lutein and unsaturated fatty acids (Ragasa *et al.*, 2014). Other compounds isolated from *A. altilis* are artoindonesianin B, artoindonesianin F and artonol B (Jagtap and Bapat 2010).

With the comparative *in vitro* anti-plasmodial activity of the leaves and stem bark and the *in vivo* antimalarial activity of the stem bark in combination with other antimalarial plants also reported (Boyom *et al.*, 2009; Adebajo *et al.*, 2013), it is imperative that this be supported with the bioassay–guided *in vivo* antimalarial evaluation. This is with the aim of isolating and identifying the chemosuppressive antimalarial constituents of the plant (Rukunga and Simons, 2006).

## Materials and Methods

### Plant Material and Extraction

The stem bark of *A. altilis* was collected in December, 2010 from the main gate and staff quarters of the Obafemi Awolowo University, Ile-Ife, Nigeria and authenticated by Prof. H C. Illoh of the Botany Department, Faculty of Science. Voucher specimens, IFE16545 were deposited at the University and Faculty of Pharmacy Herbarium, Ile-Ife, Obafemi Awolowo University, Ile-Ife respectively. A 1.70 kg of the air dried and powdered stem bark was exhaustively extracted with 70% ethanol, filtered and concentrated *in vacuo* to yield 170 g (10.0 % w/w) ethanolic extract.

### Acute toxicity testing

The acute toxicity of the resulting ethanolic extract was carried out in two phases: In the first phase, three groups of three animals each were administered orally with doses of 10, 100, 1000 mg /kg body weight respectively. These were observed for a period of 24 hours for mortality. If no mortality was observed, then three groups consisting of one animal per group were given 1600, 2900 and 5000 mg/kg body weight of the extract respectively in the second phase and observed for mortality: The lowest dose that produced mortality (**D_100_**) and the highest that produced no mortality (**D_0_**) were recorded. The LD_50_ was calculated from the formula: **LD_50_** = **√ (D_0_ x D_100_**) (Lorke, 1983).

### *In vivo* antimalarial assays

#### (i) Animals and Parasites

The chloroquine-sensitive *Plasmodium berghei berghei* NK65-infected donor mouse with rising parasitaemia was obtained from the Institute of Advanced Medical Research and Training, University College Hospital, Ibadan. The donor mouse was euthanized and blood obtained by cardiac puncture. The blood was diluted with normal saline to contain a standard inoculum of 1x10^7^ infected erythrocytes in 0.2 mls of diluted blood. This was administered intraperitoneally to Swiss albino mice weighing between 18 and 22g. The parasite was subsequently maintained by serial passaging in mice and by close monitoring of the parasitaemia level (Peters, 1965).

#### (ii) The four-day suppressive test

The *in vivo* antimalarial activities of the ethanol extract and fractions were determined using the four-day suppressive test (Peters, 1965). Experimental Swiss albino mice that have been allowed to acclimatize for at least 10 days earlier were inoculated with the *Plasmodium* parasite and randomized into different groups of five mice each as appropriate for each experiment including the negative (distilled water) and the positive control (CQ, 10 mg/kg). These were administered with the various doses of the extracts or fractions orally 2 hours after inoculation (D_o_) and repeated daily for the following three days (D_1_, D_2_, D_3_). On the fifth day (i.e., D_4_), blood was withdrawn from the tail of each mouse and the level of parasitaemia determined. The mice were further observed for 28days, for mortality, from the day of drug administration.

#### (iii) Average percentage parasitaemia, percentage chemosuppression and median effective doses

The parasitaemia level in each mouse as percentage parasitaemia was determined by counting the number of parasitised (NPE) and unparasitised (NUE) red blood cells for each of ten fields in a blood smear view under the oil immersion objective of a microscope and estimating from the formula:100(NPE)/(NPE + NUE). The average of these, for 5 mice were calculated to give the average percentage parasitaemia per dose (Peters, 1965).

The Percentage (%) chemo-suppression for each dose was afterwards calculated from the average percentage parasitaemia using the formula: APU-APT x 100/ APU where APU is average percentage parasitaemia in the untreated mice and APT is average percentage parasitaemia in the treated mice (Peters, 1965). The median effective doses, ED_50_ and ED_90_ values, which are measures of the antimalarial activity, are the doses required to reduce parasitaemia in infected mice by 50 and 90 %, respectively and were determined as forecasted from a plot of the percentage chemosuppression against the test dose using a Microsoft Excel 2007 programme.

#### (iv) Survival Times and Percentage survivors

The mice for each of the above experiments were observed daily for 28 days for mortality from the day of drug administration (Peters, 1965). The average numbers of days for which the mice survived per group were determined and recorded as mean ± SEM. The percentage survivor (PS) was recorded as a percentage of total number of animals per group that gave survival times greater than or equal to the average survival time obtained for the group. It was calculated using the formula: N_S_ /N_T_ X 100 where N_S_ is the number of animals with survival times greater than or equal to the average group survival time and N_T_ is the total number of animals per group.

### *In vivo* Antimalarial Assays of the ethanolic extract of *A. altilis*

The ethanolic extract was tested for antimalarial activities using the four-day test described in **section (ii) above**. Briefly, thirty (30) infected mice were randomized into six groups of five (5) each to be administered with doses 25, 50, 100 and 200 mg/kg of ethanolic extract (groups I-IV), chloroquine,10 mg/kg, group V as the positive control and distilled water (group VI as the negative control). These were given orally 2 hours after inoculation (D_o_) and repeated daily for the next three days (D_1_, D_2_, D_3_). On the fifth day (i.e., D_4_), the level of parasitaemia was determined for each mouse by withdrawing blood from the tail of each of the animals. The percentage (%) chemo suppression for each dose was afterwards calculated as in section **(iii**) above. The ED_50_ and ED_90_ values were forecasted from the percentage chemosuppression value for each dose using the Microsoft Excel 2007 programme. The mice were further observed for 28 days after drug administration for mortality and the survival times and percentage survivors determined as in Section **(iv**) above.

### Purification of the Stem-bark of *A. altilis* Extract

### Pre-column purification

The *A. altilis* stem bark extract (170 g) was adsorbed on 70-230 mesh silica gel (70.0 g) and air-dried to obtain a free-flowing consistency. This was packed into a sintered Buchner funnel and eluted successively under pressure with n-hexane (1500 ml) dichloromethane (2500 ml), ethyl acetate (2500 ml), and n-butanol (300 ml), and water (300 ml). Thin-Layer Chromatography was used to monitor the elution which was exhaustive at each stage before changing the solvents. The resulting fractions were concentrated in vacuo to give the n-hexane, AAH (9.63 g), AAD dichloromethane (18.66 g), AAE ethyl acetate (18.99 g), AAB n-butanol (10.59g), and AAQ aqueous (29.45 g) fractions respectively. The yields were calculated.

**Figure 1:**
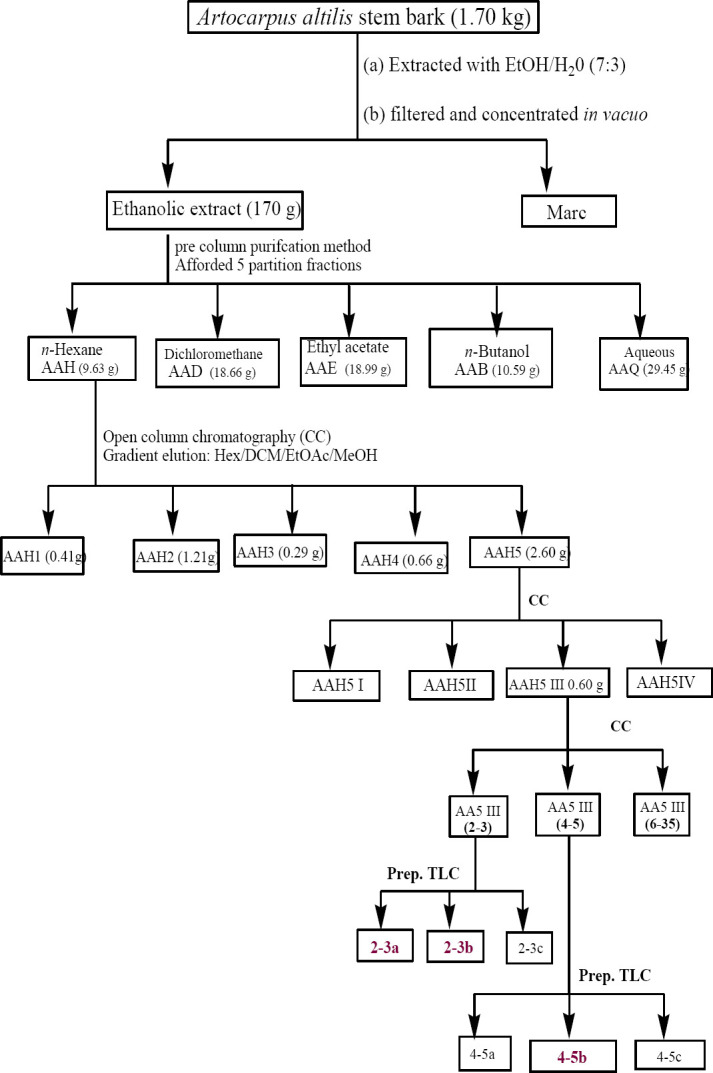
Flow Chart for the Extraction and Fractionation of *Artocarpus altilis* Stem Bark

### *In vivo* Antimalarial Assays of the partition fractions of *A. altilis*

The five (5) partition fractions, *n*-hexane, dichloromethane, ethyl acetate, *n*-butanol, and aqueous were separately tested for antimalarial activities at doses 12.5, 25.0 and 50 mg/kg using the four-day test described in Section **(ii)** above. The percentage (%) chemo suppression for each dose was afterwards calculated as in section **(iii**) above. The ED_50_ and ED_90_ values, the survival times and percentage survivors were determined as above in Section **(iii)** and **(iv**) above.

### Column Chromatography of the most active n- hexane fraction

The most active n-hexane (AAH) fraction (8 g) was adsorbed on 21 g silica gel (mesh 70-230) and subjected to further purification using column chromatography packed dry, with 150 g silica gel (70- 230 mesh). The adsorbed fraction was gently layered on the packed column and the elution was carried out with gradient mixtures of *n-*hexane, dichloromethane, ethyl acetate and methanol and the fractions collected were pooled together, based on TLC profile in solvent systems *n*-hexane-CH_2_Cl_2_ 4: 6, as follows into **AAH1 (**1-17, n-hexane,100mls; n-hexane :CH_2_Cl_2_ 6:4, 100mls; 1:1, 70ml, 0.41g**), AAH2** (18–32; n-hexane: CH_2_Cl_2_ 1:1, 30mls, n-hexane: CH_2_Cl_2_ 4:6, 50mls; n-hexane : CH_2_Cl_2_ 2: 8, 200mls; n-hexane : CH_2_Cl_2_ 1:9, 40mls; 1.21 g), **AAH** (33–46, n-hexane: CH_2_Cl_2_ 1:9, 60mls; n-hexane : CH_2_Cl_2_ 100%, 250mls; 0.29 g), **AAH4** (47–56, CH_2_Cl_2_–EtOAc 9 : 1 100mls; CH_2_Cl_2_–EtOAc 8 : 2, 100mls, CH_2_Cl_2_–EtOAc 6 : 4, 25mls; 0.66 g) and **AAH5 (**57–76, CH_2_Cl_2_–EtOAc 6 : 4, 125 mls, CH_2_Cl_2_–EtOAc 4 : 6, 100mls, CH_2_Cl_2_–EtOAc 2 : 8, 100mls; EtOAc 100%, 150mls; MeOH, 100%, 200mls, 2.60 g**)**.

### *In vivo* antimalarial assays of the bulked column fractions of the n-hexane fraction

The pooled column fractions AAH1, AAH2, AAH3, AAH4 and AAH5 of the *n*-hexane partition fraction of *A*. *altilis* stem bark were subjected to the *in vivo* antimalarial four-day suppressive (Peters, 1965), as in Section **(ii)** above. The percentage (%) chemo suppression for each dose was afterwards calculated as in section **(iii**) above. The ED_50_ and ED_90_ values, the survival times and percentage survivors were determined as above in Section **(iii)** and **(iv**)above.

## Statistical Analysis

The antimalarial activities of *A. altilis* extract, partition and bulked column fractions of the most active *n*-hexane fraction were shown by comparing their percentage chemosuppression and ED_50_, ED_90_ with those of the positive and negative control and with each other by subjecting the values to statistical analysis using ANOVA followed by Dunnett and Bonferroni t-test as the post-hoc tests. P < 0.05 was considered as significant.

### Purification of the most active column fraction AAH5

The most active column fraction **AAH5** **(2.0 g)** was purified to give **AAH5** I-IV of which **AAH5** III was chosen for further purification. Therefore, 600.0 mg of **AAH5** III was adsorbed with 500.0 mg of silica gel and eluted with gradient concentrations of dichloromethane and ethyl acetate on a silica gel column packed with dichloromethane, as follows: **(1**, CH_2_Cl_2_: EtOAc, 10:0, 15.0 mls,) **(2**, CH_2_Cl_2_: EtOAc, 10:0, 15mls) **(3**, CH_2_Cl_2_: EtOAc, 10:0, 15.0 mls) **(4**, CH_2_Cl_2_:EtOAc, 10:0, 15.0 mls) **(5**, CH_2_Cl_2_:EtOAc, 10:0, 15.0 mls) **(6**, CH_2_Cl_2_:EtOAc, 10:0, 15.0 mls, **7-9** CH_2_Cl_2_:EtOAc, 10:0, 45.0 mls)**, (10-13**, CH_2_Cl_2_:EtOAc, 10:0, 60.0 mls,) **(14-15**, CH_2_Cl_2_:EtOAc, 10:0, 30.0 mls,) **(16-17**, CH_2_Cl_2_:EtOAc, 9:1, 30.0 mls, ) **(19**, CH_2_Cl_2_:EtOAc, 8:2, 15.0 mls,) **(20**, CH_2_Cl_2_:EtOAc, 7:3, 15.0 mls,) **(21-22**, CH_2_Cl_2_:EtOAc, 6:4, 30.0 mls,. **(23-25**, CH_2_Cl_2_: EtOAc, 1:1, 45.0 mls **(26-29**, CH_2_Cl_2_: EtOAc, 3:7, 45.0 mls **(30-32**, CH_2_Cl_2_: EtOAc, 2:8, 45.0 mls **(33-35**, CH_2_Cl_2_: EtOAc, 1:9, 45.0 mls) and bulked as follows: AAH5 **III 2-3 (**brownish yellow oil, 0.15g**);** AAH5 **III 4-5 (**crystalline light-yellow oil, 0.17g**); 6-19** (deep oily yellow crystal, 0.14g)**; 22-30** (yellowish brown oil, 0.04g)**, 30-35 (**dark brown oily substance 0.02g**).**

Both AAH5 **III** 2-3 and AAH5 **III** 4-5 were subjected to PTLC, (0.75 mm, CH_2_Cl_2_: MeOH, 9:1) to yield 3 bands each (AAH5 III 2-3 a (16 mg), AAH5 III 2-3 b (13 mg) and AAH5 III 2-3c (1 mg), AAH5 III 4-5 a (1 mg), AAH5 III 4-5b (14 mg), AAH5 III 4-5c (1 mg) from which (AAH5 III 2-3 a (16 mg); **A1**; AAH5 III 2-3 b (13 mg); **A2**; and AAH5 III 4-5b (14 mg); **A3** were chosen for GC-MS analysis.

### Gas Liquid Chromatographic Analysis

Gas Liquid Chromatographic (GLC) separation was performed on a Gas chromatography (Agilent, USA) hyphenated to a mass spectrophotometer (5957C) with triple axis detector equipped with an auto injector (10 μL syringe) with Helium gas as carrier. All chromatographic separation were performed on a capillary column, specification 19091S-433 HP-5MS, dimensions: 30 m x 250 μm x 0.25 μm, treated with phenyl methylsilox and operated at a constant flow rate of 1.5 mL/min of helium gas with other conditions as follows: EI (ion source temperature), 250 °C, interface temperature 300 °C, pressure 16.2 psi, out time 1.8 mins; 1.0ml injector in the split mode with a split ratio 1:50 and an injection temperature of 300 °C. The oven temperature was held for 5.0 min at an initial temperature of 35 °C and programmed to increase to 150 °C at 4 °C/min, held for 2 min and later increased to 250 °C at 20° C/min and finally held isothermally for 5 minutes, giving a total run time of 47.5 mins. Transfer line temperature was set to 34 °C and post run temperature was to 325 °C for 10 min. The data solution software supplied was used to control the system and acquire the data. The separated constituents were passed to the detector which recorded the emergence of the constituents as peaks with a retention time. The percentage compositions of the compound in the entire sample were computed from the peak areas automatically generated by the machine. The results were recorded as retention time against percentage composition in the original sample.

### Gas Chromatographic–Mass Spectrometric (GC-MS) Analysis of A1, A2 and A3

C-MS analysis was performed on **A1,** AAH5 III 2-3a**;** **A2,** AAH5 III 2-3b and **A3**, AAH5 III 4-5b as stated above. Samples were prepared and injected into the GC-MS machine and the result acquired as peaks with respective retention times.

Data handling was done using GC-MS solution software. The identities of the components were assigned by comparing their retention times with those of the standard spectra from NIS

### Ethical Approval

The protocol used for this study was approved by the Board of Postgraduate College, OAU with the Registration Number PHP/H/10/11/1017. Principles of laboratory animal care” (NIH publication No. 85-23, revised 1985) were followed, as well as specific national laws where applicable.

## Results

### Acute toxicity test

No mortality was observed at >5000mg/kg it means that the extract is orally safe (Lorke, 1983).

**Figure 2:**
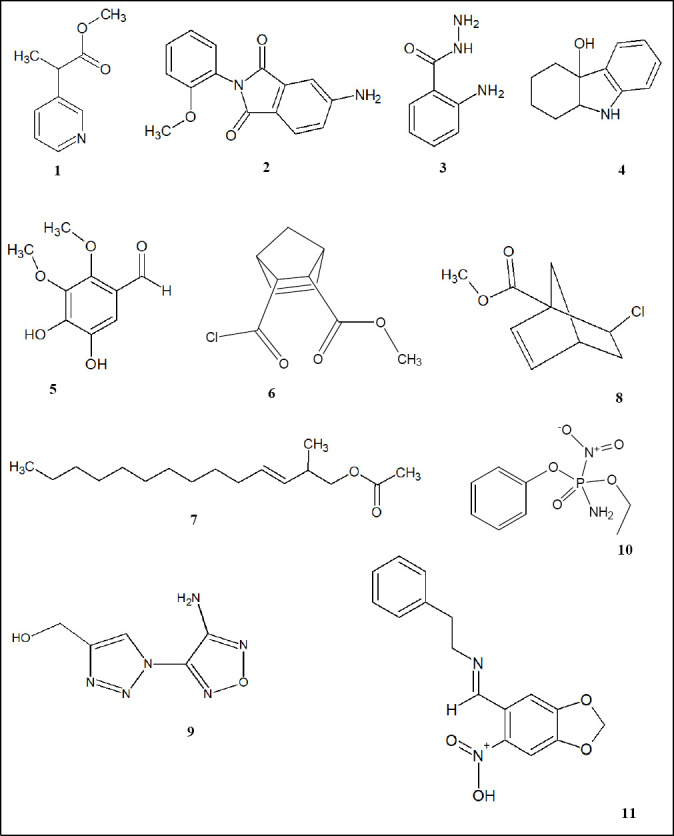
Structures of the Major Compounds Identified in the Purified Fractions

## Discussion and Conclusion

Medicinal plants with claims of antimalarial activities are potential sources of antimalarial agents (Iwalewa *et al.*, 2008). Therefore, research efforts have been directed at verifying such claims by scientists using multifarious approaches such as activity-directed *in vivo* investigation of plant extracts on rodent parasite infected models in order to establish antimalarial action (Carvalho *et. al*., 1991). Some plants such as *Quassia amara*, *Quassia undulate*, *Zingiber officinale*, *Acacia nilotica*, *Xylopia aethiopica* and *Artemisia maciverae* have been screened through such model for antimalarial activities (Ajaiyeoba *et al.*, 1999).

Incidence of renal and hepatic toxicity has been recorded with the ingestion of the medicinal herbs particularly at high dose (Pieme *et al.*, 2006), hence the need for evaluation of their safety and efficacy profile (Ogbonnia *et al.*, 2010). The stem-bark of *Morinda lucida* was reported to be extremely toxic (Ajaiyeoba *et al.*, 2006). Therefore, in this study, toxicity evaluation preceded anti-plasmodial testing. The LD_50_ values are the usual indices of toxicity potentials of plant extracts and also a means of determining the safe doses to be used for testing the activities of extracts. The LD_50_ determined for the ethanolic extract of the *A. altilis* stem-bark was above 5,000mg/kg. This implies that the oral use of *A. altilis* extract is safe (Lorkes, 1983) and the doses 0-800 mg/kg body weight chosen for the ethanolic extract is justified.

The *in vitro* and *in vivo* antimalarial tests are established models for assessing antimalarial activities and both should be performed to justify the antimalarial activities of a plant extract or fraction (Aladesanmi *et al*, 1988; Rukunga and Simons 2006, Adebajo *et al.*, 2013). The *in vitro* antimalarial property of *A. altilis* has been reported (Boyom *et al.*, 2009*); in vivo* activity was therefore attempted to complement and justify antimalarial efficacy of this plant. *P. berghei*, a rodent parasite was chosen for the *in vivo* chemosuppressive activity in the four- day antimalarial test (Peters, 1965). This method was also used by Ebiloma *et al.*, 2011 in the investigation of the antimalarial activity of *Morinda lucida* (Benth) against erythrocytic stage of mice infected with chloroquine sensitive *Plasmodium berghei* NK-65.

### Antiplasmodial activities

The percentage chemosuppression derived from the percentage parasitaemia is employed as a suitable measure of anti-malarial activity especially in the *in vivo* experiment (Ebiloma *et al.*, 2011). The dose-dependent percentage chemosuppression of the ethanolic extract up to 200 mg/kg (Table1) was comparable to chloroquine, an established drug used in the treatment of malaria (Aliyu, 2013). This activity of *A. altilis* may be due to a synergistic combination of some constituents present in the extract. The effect may also likely be better against resistant parasite strains than chloroquine. However, solvent partitioning of the ethanolic extract into different solvents was to profile the chemical constituents according to their polarity. The significantly lower ED_50_ and ED_90_ values of *n*-hexane partition fraction compared to that of ethyl acetate and butanol (Tables 2 and 3) informed its choice as the most active partition fraction.

**Table 1:** Average percentage parasitaemia and percentage chemosuppression in the *in vivo* chemosuppressive antimalarial activities of ethanol *Artocarpus altilis* stem-bark extract in mice

Doses (mg/kg)	% Parasitaemia	% Chemosuppression
NC	8.87±0.5^b^	0.00±0.0^a^
100	1.20±0.6^a^	86.47±5.2^b^
200	0.43±0.1^a^	95.15±4.0^b^
400	1.18±0.3^a^	86.69±6.3^b^
800	1.63±0.2^a^	81.60±6.5^b^
CQ	0.27±0.1^a^	98.97±2.3^b^

**Keys:** Data show the mean ± SEM, *n* = 5: NC (negative control): Tween 80 in normal saline; CQ = Chloroquine (10 mg/kg). Only values with different superscripts of alphabets within columns are significantly different (*p* < 0.05, one-way analysis of variance followed by the Student–Newman–Keul’s post hoc test).

**Table 2:** Percentage chemosuppression elicited at different doses by the partitioned fractions of the ethanol extract of the *Artocarpus altilis* stem-bark in an *in vivo* antimalarial activity test

Doses	% Chemosuppression by partitioned fractions ± SEM				
	AAH	AAD	AAE	AAB	AAQ
NC	0.00±0.0^a^	0.00±0.0^a^	0.00±0.0^a^	0.00±0.0^a^	0.00±0.0^a^
25	48.29±1.1^b^	10.56±1.8^b^	3.06±0.8^a^	40.51±2.2^d^	45.31±0.7^c^
50	50.71±1.5^b^	23.82±3.6^c^	6.36±2.5^a^	27.25±1.8^c^	62.19±0.8^d^
100	70.23±0.8^c^	10.75±3.8^b^	48.79±3.4^c^	66.26±0.9^e^	38.55±2.7^b^
200	70.83±1.0^c^	28.81±3.6^c^	39.73±3.1^b^	14.91±4.2^b^	46.78±4.7^c^
PC	82.62±0.9^d^	82.62±0.9^d^	82.62±0.9^d^	82.62±0.9^f^	82.62±0.9^e^

**Keys:** **AAH** = N-hexane; **AAD** = Dichloromethane; **AAE** = Ethyl acetate: **AAB** = Butanol; **AAQ**: Aqueous partitioned fractions. Data show the mean ± SEM, *n* = 5. NC (negative control): Tween 80 in normal saline. Only values with different superscripts of alphabets within columns are significantly different (*p* < 0.05, one-way analysis of variance followed by the Student–Newman–Keuls’ post hoc test).

**Table 3:** Median Effective Doses (ED_50_ and ED_90_) values of the partitioned fractions of *Artocarpus altilis* stem-bark extract in *in vivo* chemosuppressive antimalarial activities test.

FRACTIONS	ED_50_	ED_90_
**AAH**	79.14± 1.9^a^	162.43±1.8^c^
**AAD**	215.59±5.2^d^	358.58±5.2^e^
**AAE**	160.46±3.0^c^	171.87±3.0^d^
**AAB**	81.42±3.0^a^	94.12±3.0^a^
**AAQ**	90.85±3.3^b^	146.17±3.3^b^
**EEE**	227.17±0.3^e^	372.96±0.3^f^

**Keys:** **AAH** = N- hexane; **AAD** = Dichloromethane; **AAE** = Ethyl acetate: **AAB** = Butanol; **AAQ** = Aqueous partitioned fractions; EEE = Methanol extract. Data show the mean ± SEM, *n* = 5. NC (negative control): Tween 80 in normal saline. ED_50_ and ED_90_ are doses of the extracts that gave 50 % and 90% activity respectively. Only values with different superscripts of alphabets within columns are significantly different (*p* < 0.05, one-way analysis of variance followed by the Student–Newman–Keuls’ post hoc test).

However, dichloromethane partition fraction gave the lower percentage of chemosuppression at 50 mg/kg dose and higher ED_50_ values compared to the aqueous partition fractions. The order of activity therefore for the partitioned fractions is AAH>AAB>AAQ>AAE>AAD. All were more active than the ethanol extract. The *n*-hexane fraction was therefore purified by column chromatography and the five pooled fractions, AAH1, AAH2, AAH3, AAH4 and AAH5 obtained were further subjected to anti-malarial testing.

All the pooled column fractions elicited a dose-dependent percentage chemosuppression (Table 5) but comparable ED_50_ and ED_90_ except the AAH2 (Table 6), though were all significantly better in activity than those of the ethanolic extract and *n*-hexane fraction (Table 1). The order of activity for the pooled column fractions is AAH1=AAH3=AAH4=AAH5>AAH2. AAH5 was chosen for purification by a further column chromatography.

**Table 4:** Survival time and percentage survivor (in parenthesis) of mice of the Partitioned fractions of *Artocarpus altilis* stem-bark using a-4-day test.

Doses	Survival times by partitioned fractions ± SEM				
	AAH	AAD	AAE	AAB	AAQ
NC	6.0 ±0.8^a^ (20)	6.0 ±0.8^a^ (20)	6.0 ±0.8^a^ (20)	6.0 ±0.8^a^ (20)	6.0 ±0.8 ^a^ (20)
25	20.75±2.7^b^ (20)	8.80±2.7^b^ (60)	13.00±1.0^a^ (60)	10.60±1.6 ^a^ (60)	12.4±0.4^a^ (20)
50	12.60±2.2^a,b^ (60)	12.40±2.2^b^ (60)	10.60±0.9^a^ (60)	10.60±1.7^a^ 60)	15.75±2.1^a^ (40)
100	17.60±4.7^a,b^ (40)	7.00±1.5^a^ (60)	11.40±1.8^a^ (40)	11.80±0.6^a^ (60)	10.8±0.8^a^ (80)
200	15.00±4.3^a,b^ (60)	12.80±5.2^b^ (40)	11.80±2.4^a^ (60)	12.80±1.7^a^ (40)	13.4±1.3^a^ (40)
PC	20.25±3.5^b^ (60)	20.25±3.5^b^ (60)	20.25±3.5^b^ (60)	20.25±3.5^b^ (60)	20.25±3.5^b^ (60)

**Keys:** **AAH** = N- hexane; **AAD** = Dichloromethane; **AAE** = Ethyl acetate: **AAB** = Butanol; **AAQ** = Aqueous partitioned fractions. Data show the mean ± SEM, *n* = 5. NC (negative control): Tween 80 in normal saline. Only values with different superscripts of alphabets within columns are significantly different (*p* < 0.05, one-way analysis of variance followed by the Student–Newman–Keuls’ post hoc test).

**Table 5:** Percentage chemosuppression elicited at different doses by the chromatographic fractions of the methanol extract of the *Artocarpus altilis* stem-bark in an *in vivo* antimalarial activity test

Doses	% Chemosuppression by chromatographic fractions ± SEM				
	AAH1	AAH2	AAH3	AAH4	AAH5
**NC**	0.00±0.0^a^	0.00±0.0^a^	0.00±0.0^a^	0.00±0.0^a^	0.00±0.0^a^
**12.5**	50.12±0.3^b^	48.21±0.1^b^	46.41±0.2^b^	64.11±0.2^b^	61.60±0.2^b^
**25.0**	78.35±0.3^d^	56.58±0.1^c^	77.03±0.2^c^	70.69±0.2^c^	66.75±0.2^c^
**50**	70.93±0.2^c^	61.72±0.2^d^	81.22±0.1^d^	73.44±0.2^d^	80.74±0.1^d^
**PC**	89.95±0.1^e^	89.95±0.1^e^	89.95±0.1^e^	89.95±0.1^e^	89.95±0.1^e^

**Keys: AAH1-5** = successive chromatographic fractions of AAH. Data show the mean ± SEM, *n* = 5. NC (negative control): Only values with different superscripts of alphabets within columns are significantly different (*p* < 0.05, one-way analysis of variance followed by the Student–Newman–Keuls’ post hoc test).

**Table 6:** Median Effective Doses (ED_50_ and ED_90_) values of the chromatographic fractions of the n-hexane partitioned fraction of *Artocarpus altilis* stem-bark in an *in vivo* chemosuppressive antimalarial activities test.

FRACTIONS	ED_50_	ED_90_
**AAH1**	22.31±1.2^a^	40.89±1.1^a^
**AAH2**	27.37±1.3^b^	51.6±2.2^b^
**AAH3**	21.6±0.9^a^	37.49±4.5^a^
**AAH4**	21.07±0.5^a^	39.33±1.1^a^
**AAH5**	20.9±0.5^a^	40.78±0.9^a^
**AAH**	79.14± 1.9^c^	162.43±1.8^c^

**Keys:** **AAH1-5** = successive chromatographic fractions of AAH. Data show the mean ± SEM, *n* = 5. NC (negative control): Tween 80 in normal saline. ED_50_ and ED_90_ are doses of the extracts that gave 50 % and 90% activity respectively. Only values with different superscripts of alphabets within columns are significantly different (*p* < 0.05, one-way analysis of variance followed by the Student–Newman–Keuls’ post hoc test).

### Survival times and Percentage Survivors (PS)

The n-hexane partition fraction, at 25mg/kg, elicited survival times that was significantly different from that elicited by negative control and comparable to that of chloroquine, the positive control drug (Table 4). Also, at the same dose of 25mg/kg, n-hexane produced significantly (p<0.05) higher survival times in mice than other partition fractions. However, the survival times elicited by all the doses of the AAD partition fraction except that of 100mg/kg were comparable to that of the positive control. All the doses of the ethyl acetate fraction just like the butanol fraction produced lower survival time values which were all comparable to the negative control. For the aqueous fraction, only 50 mg/kg gave values that were comparable (p>0.05) to CQ, others produced values which were significantly lower than CQ. Therefore, the order of activity using the survival time for the partition fraction is AAH>AAD>AAQ>AAB= AAE. The better survival times elicited by the n-hexane partition fraction (AAH) than other fractions further confirm it as the most active partition fraction.

The survival time produced by AAH1 just like AAH3 at 12.5mg/kg dose was significantly (p<0.05) higher than that produced by the positive control. The values were also comparable to that elicited by those of 25 and 50mg/kg doses. Also, the percentage survivor (PS) of 60% given by AAH1 and AAH3 at 12.5mg/kg corroborate it’s significantly (p<0.05) higher activity than the positive control with 40% PS. Also, the 80% PS given by 50mg/kg, the highest tested dose for AAH3 attested to its better activity than AAH1 while AAH2 and AAH4 gave comparable survival time values at all the doses tested to the negative control. The AAH5 at 12.5mg/kg gave significantly (p<0.05) higher survival times than the other doses including the negative control. It also showed comparable activities to the positive control drug and a PS of 40% at all its tested doses. Therefore, the order of activity using the survival time for the column bulked fractions is AAH1>AAH3>AAH5>AAH2=AAH4. The significantly high percentage chemosuppression, the low ED_50_ and ED_90_ values coupled with the very high survival time of the *n*-hexane partitioned fraction made it the choice for further purification. The *n*-hexane extract and purified bulked fractions of *K. grandifoliola* have been reported to give 91 % chemosuppression *in vivo* in mice and IC_50_ values of 1.4 μg/mL (for multiple-drug resistant clone) or 0.84 μg/mL (for Nigerian *P. falciparum* isolates) (Agbedahunsi *et al* ., 1998). Also, the *n*-hexane fraction of *H. madagascariensis* exhibited the highest suppressive activity of 93.94 % at 40 mg/kg among other fractions (Iwalewa, *et al.*, 2008). Though all the bulked column fractions of the most active *n*-hexane partition fraction, except AAH2 gave comparable (p>0.05) ED_50_ and ED_90_ values, AAH1 and AAH3 gave comparably higher survival times hence suitable candidate fractions for further purification. However, the thin-layer chromatography (TLC) of all the column fractions showed the respective number of visible spots and weight as follows: AAH1 (8 spots, 0.41g), AAH2 (13 spots, 1.21 g), AAH3 (12 spots, 0.29 g), AAH4 (6 spots, 0.66 g), AAH5 (4 spots, 2.60 g). Therefore, further purification works should be concentrated on these bulked column fractions particularly **AAH1** and **AAH3** with high antiplasmodial activities. However, **AAH5** was chosen for further purification because of its weight, the fewer number of TLC spots and the highest survival times (Table 7). It was purified, therefore, by a further column chromatography to give AAH5 I, AAH5 II, **AAH5III,** AAH5 IV and AAH5 V from which AAH5 III was further subjected to repeated PTLC to give **A1** (AAH5 **III** 2-3 a (16mg), **A2** AAH5 **III** 2-3 b (13mg) and **A3** AAH5 **III** 4-5b (14mg) which were chosen for GC-MS analysis.

**Table 7:** Survival time and percentage survivor (in parenthesis) of mice of the chromatographic fractions of *Artocarpus altilis* stem-bark Ethanolic Extract using the 4-day test.

Doses	Survival times by chromatographic fractions ± SEM				
	AAH1	AAH2	AAH3	AAH4	AAH5
NC	14.00±2.0^a^ (20)	14.00±2.0^a^ (20)	14.00±2.0^a^ (20)	14.00±2.04^a^ (20)	14.00±2.0^a^ (20)
12.5	21.40±1.5^c^ (60)	17.80±1.8^a^(40)	20.20±1.3^c^ (60)	16.80±0.86^a^ (60)	22.60±1.3^b^ (40)
25.0	15.00±1.5^b^ (80)	16.40±1.1^a^ (40)	14.4±0.5^b^ (40)	16.4±0.81^a^ (40)	17.20±0.8^a^ (40)
50	16.80±1.9^b^ (80)	15.80±2.7^a^ (40)	17.80±1.2^b^ (80)	17.20±2.1^a^ (40)	18.62±2.7^a^ (40)
PC	18.20±0.9^a^ (40)	18.20±0.9^a^ (40)	18.20±0.9^a^ (40)	18.20±0.9^a^ (40)	18.20±0.9^a^ (40)

**Keys:** **AAH1-AAH5** = successive column fractions of AAH. Data show the mean ± SEM, *n* = 5. NC (negative control): Tween 80 in normal saline. Only values with different superscripts of alphabets within columns are significantly different (*p* < 0.05, one-way analysis of variance followed by the Student–Newman–Keuls’ post hoc test).

**Table 8:** Gas-Chromatographic Analysis of the isolates from purified fractions of *Artocarpus altilis* Stem-Bark ethanolic Extract

CI	CODES	NAME OF COMPOUNDS	M/W	FORMULA	CAS NUMBER	R_t_ (min)	PEAK AREA
1	A1 (AAH5 III 2-3a)	Pyridin-3-yl 2-methylbutanoate	179.22	C_10_ H_13_ NO_2_	1000350-90-2	17.05	2.04
2	A1 (AAH5 III 2-3a)	2H-Isoindole-1,3(1H,3H)-dione,5 amino-2-(2-methoxyphenyl)	268.08	C_15_ H_12_ N_2_ O_3_	000622-37-7	14.927	2.42
3	A1 (AAH5 III 2-3a)	2-Aminobenzoyl hydrazide	151.17	C_7_ H_9_ N_3_ O	001904-58-1	14.664	2.07
4	A2 (AAH5 III 2-3b)	1,2,3,4-tetrahydro-carbazol-4a-ol	187.09	C_12_ H_13_ NO	312315-76-7	9.234	2.76
5	A2 (AAH5 III 2-3b)	4,5-dihydroxy-2,3-di- methoxy benzaldehyde	198.05	C_9_ H_10_ O_5_	-	11.677	4.25
6	A2 (AAH5 III 2-3b)	Ethyl 3-(chlorocarbonyl)bicyclohept-5-ene-2-carboxylate	216.08	C_10_ H_11_ ClO_3_	69610-44-2	11.893	2.39
7	A2 (AAH5 III 2-3b)	E-2-Methyl-3-tetradecen-1-ol acetate	268.24	C_17_ H_32_ O_2_	-	12.969	3.97
8	A3 (AAH5 III 4-5b)	Bicyclo[2.2.1]hept-2-ene-1-carboxylic acid, 6-chloro-, methyl ester, endo	186.04	C_9_ H_11_ ClO	-	11.048	3.27
9	A3 (AAH5 III 4-5b)	1-(4-aminofurazan-3-yl)-1,2,3-triazole-4-methanol	182.10	C_5_ H_6_ N_6_ O_2_	-	9.609	3.23
10	A3 (AAH5 III 4-5b)	Phosphoramidic acid, ethyl p-nitro phenyl diester	246.08	C_8_ H_12_ N_2_ PO_5_	-	10.604	2.82
11	A3 (AAH5 III 4-5b)	4-Dehydroxy-N-(4,5-methylene-dioxy)-2-nitrobenylidene) tyramine	298.29	C_16_ H_14_ N_2_ O_4_	-	10.629	3.35

**Key:** Compound Identified (CI); Molecular Weight (M/W); Retention time (R_t_)

### Identification of the constituents of the most active PTLC bands isolates after GC-MS

The hyphenated technique of Gas chromatography-mass spectrometry (GC–MS) is a valuable tool in natural product research, assisting in the separation and identification of chemical components of complex volatile mixtures found in petrochemicals and volatile oils of plants in many natural product researches (Li *et al.*, 2009; David *et al*, 2015), and organic extracts (Kimaru and Nguyen, 2014; Gomathi *et al*. 2015). It is known for its high-resolution and separations of isomeric structurally similar mono- and sesqui-terpenes that are the main constituents of plant essential oils. In practice, isolation is only mandatory when a component is suspected to be new (Kimaru and Nguyen, 2014). The use of electron ionisation-mass spectrometry (EI-MS) produces distinctive mass spectral fragmentation patterns, which when compared with stored EI spectra in the computer library, may help in identifying the separated components. In hyphenation with GC therefore, using the R_t_, MS library and co-injection of pure isolates (reference standards), most peaks of the GC analyses of volatile oils have been conclusively identified while suggestions were made for the unknown components (Onayade and Adebajo, 2000; Li *et al.*, 2009). The reversed phase column HP-5MS (5 % phenyl methyl silicone) that was used, should elute the most polar constituents first (Molnar and Horvath, 1976) and this knowledge would also assist in the identification of the resolved components.

GC-MS data obtained for AAH5 **III 2-3a,** AAH5 **III 2-3 b** and AAH5 **III 4-5b** were therefore utilized in the identification of the constituents of this plant being the isolates likely to contain the antimalarial constituents of the plant. These three isolates afforded 49, 8 and 55 peaks respectively. Six of the AAH5 **III 2-3a** peaks have > 2% peak area, the others are <2%. That of AAH5 III 2-3 b gave 4 peaks that were >3% peak area while 12 such peaks with sizes >2%. This implies that these various compounds occur in very low concentrations and it will only suffice to identify the major ones. The peaks selected eventually were the most resolved and with higher concentration in the fraction. Therefore, three, four and four major peaks were characterized respectively from the isolates using their retention times and the sizes of the peaks. Hence, the following compounds were identified as the major constituents of the plant after comparing their retention times and mass spectra data with that of standard compounds. This identified the respective compounds with retention times and peak areas as components of the resolved mixtures respectively as follows:

**AAH5 III 2-3a: (3 compounds)** **(i)** (2-Pyridin-3-yl methylbutanoate (1000350-90-2, 17.053, 2.04); **(ii)** 2H-Isoindole-1, 3 (1H, 3H)-dione, 5 amino-2-(2-methoxyphenyl (000622-37-7; 14.927, 2.42), **(iii)** 2-Aminobenzoyl hydrazide (001904-58-1; 14.664, 2.07) ;

**AAH5 III 2-3b:** (**4 compounds**) **(i)** Carbazol-2-ol, 1, 2, 3, 4-tetrahydro-: (312315-76-7, 9.234, 2.76), **(ii)** Benzaldehyde, 4, 5-dihydroxy-2, 3-dimethoxy (11.6767, 4.25), **(iii)** Ethyl 3-(chlorocarbonyl) bicyclohept-5-ene-2-carboxylate), (11.893 2.39); **(iv)** E-2-Methyl-3-tetradecen-1-ol acetate (12.969, 3.97);

**AAH5 III 4-5b:** **(4 compounds**) **(i)** Bicyclo [2.2.1] hept-2-ene-1-carboxylic acid, 6-chloro-, methyl ester, endo (11.048, 3.27); **(ii)** 1, 2, 3-Triazole-4-methanol, 1-(4-aminofurazan-3-yl) - : 9.609, 3.23). (**iii**) Phosphoramidic acid, ethyl p-nitro phenyl diester (10.604, 2.82) and **(iv)** 4-Dehydroxy-N-(4, 5-methylenedioxy-2-nitrobenylidene) tyramine (10.629, 3.35).

In summary, it was observed that successive purification of the original ethanolic extract (EE) enhanced anti-malarial activity at each stage; the observed antimalarial activity in the extract, partitioned and bulked column fractions being attributable to its chemical constituents.

Also, previous works have shown the anti-malarial activity of chemical constituents like alkaloids and flavonoids isolated from plants (Okokon *et al.*, 2006; Balogun *et al.*, 2009). Also the antimalarial activity observed in this study could be due to single or combined effect of these compounds (Ebiloma *et al.*, 2011). Although numerous bioactive agents have been isolated from the roots, stem bark and leaves of *Artocarpus communis*, few antimalarial studies have been reported to date (Boyom, *et al.*, 2009). The investigation of a related species (*Artocarpus integer*) led to the isolation of prenylated stilbene with an IC_50_ of 1.7 μg/ml against *Plasmodium falciparum* in culture (Boonlaksiri *et al.*, 2000).

## Conclusion

In conclusion, this study has justified the use of the stem bark of *Artocarpus altilis* for malaria therapy in traditional medicine.

### Conflict of Interest

The authors hereby declare that there is no conflict of interest associated with this article.

**List of abbreviations used:**
EE: ethanol extract of *Artocarpus altilis* stem bark;
AAH: the n-hexane partition fraction of EE;
AAD: the dichloromethane partition fraction of EE;
AAE: the ethyl acetate partition fraction of EE;
AAB: the butanol partition fraction of EE;
AAQ: the residual aqueous fraction finally remaining from EE;
AAH1: first pooled chromatographic fraction of AAH;
AAH2: second pooled chromatographic fraction of AAH;
AAH3: third pooled chromatographic fraction of AAH;
AAH4: fourth pooled chromatographic fraction of AAH;
AAH5: last pooled chromatographic fraction of AAH;
NPE: the number of parasitised red blood cells;
NUE: the number of unparasitised red blood cells;
APU: average percentage parasitaemia in the untreated mice;
APT: average percentage parasitaemia in the treated mice;
N_S_: number of animals with survival times greater than or equal to the average group survival time;
N_T_: the total number of animals per group
